# The Self-Limited Duration of Pulmonary Phthisis

**Published:** 1883-01

**Authors:** Austin Flint

**Affiliations:** Professor of the Principles and Practice of Medicine and Clinical Medicine, Bellevue Hospital, New York


THE SELF-LIMITED DURATION OF PULMONARY PHTHISIS. Read in the Section of Medicine at the Annual Meeting of the British Medical Association in Worcester, August, 1882. By AUSTIN FLINT, M.D., LL.D., Professor of the Principles and Practice of Medicine and Clinical Medicine, Bellevue Hospital, New York.

My object in this paper is to show that the pulmonary phthisis may have a self-limited duration ; that, in a certain proportion of cases, this disease ends favorably, irrespective of any appreciable extrinsic agencies, recovery taking place, provided the nature and extent of the local lessions be not such as to render them either irreparable or innocuous. If the doctrine of self-limitation as applied to phthisis be not entirely new, it has, at all events, received as yet very little consideration in medical literature and in medical practice. If the doctrine in this application be true, it has important pathological and practical bearings, to some of which I shall briefly advert.

How is self limitation to be proved as applied to phthisis or to any other disease? Facts pertaining to morbid anatomy and to therapeutics may render the application of the doctrine probable; but, evidently, positive and complete proof can only be afforded by a collection of cases in which the disease pursued its course without active interference in the way of treatment, either medicinal or hygienic, and without notable changes in habits of life, or in any of the conditions under which the patients were situated when the disease became developed. For obvious reasons these requirements for absolute proof are not easily obtained in cases of a disease like pulmonary phthisis. Yet cases involving these requirements occasionally come under medical observation. The hopeful mental state which generally accompanies phthisis sometimes leads patients to trust altogether to nature for restoration to health, and to continue their usual manner of living without any alteration. Some patients do this from a conviction that they have not a malady of sufficient consequence to claim attention, beyond, perhaps, palliative remedies ; and some from circumstances which render it difficult to do otherwise. Again, there are phthisical patients who do nothing in the way of either therapeutics or hygiene from a thorough scepticism as to the advantage of doing anything.

In 1858, I had collected a considerable number of histories of cases of phthisis, recorded during the preceding twenty years of medical practice, and I was led to examine the collection for those cases in which there had been an arrest of the disease. Twenty-four cases were in this category. The histories of these twenty-four cases were analyzed with reference to points of agreement in the management; I assumed that in the points of agreement must lie the means by which the disease had been arrested, provided these points of agreement were not equally common in other cases in which the disease was not arrested. A striking result of this analytical study was, that in a few cases no appreciable influences, either of medication, diet, or regimen, had been brought to bear on the disease; the patients took no active remedies, and continued unchanged the same habits of living as before the development of the disease. It seems a logical inference that in these cases the disease was not arrested, but that the recovery was owing to an intrinsic tendency thereto. An abridged account of the histories of these twenty-four cases was embraced in a report published in the *American Journal of Medical Sciences*, January 1858.

In 1863, I had accumulated additional cases. The number amounted to sixty-two. The cases were studied analytically in the same way as those analyzed in 1858. In seven cases, no medicinal or hygienic measures of management were employed. The recovery in four of these cases was complete. In three cases good general health had been regained and maintained for a long period, some cough and expectoration remaining. An abstract of the histories of sixty-two cases was published in the *Transactions of the New York Academy of Medicine* for the year 1863.

In 1875 were published, in a work entitled *Phthisis in a Series of Clinical Studies*, the results of an analysis of the histories of all the cases of phthisis which I had recorded during thirty-four years, the number being 670. Of these 670 cases forty-four ended in recovery. Details of the history of each of the forty-four cases are given in the work sufficiently to render evident the recovery and the correctness of the diagnosis. In addition to these forty-four cases, there were thirty-one cases in which the disease ceased to progress, and remained non-progressive for at least several months, and in most instances for several years. In these thirty-one cases, the phthisical disease was considered as having ended, complete recovery not taking place in consequence of irreparable lesions. As cases for analytical study with reference to the agencies which may have caused the arrest of the disease, these thirty-one cases of non-progressive phthisis seemed hardly less valuable than the forty-four cases which ended in complete recovery. Adding together the two groups of cases, out of the 670 recorded histories of phthisis, there were seventy-five in which the disease either ended in complete recovery or remained for a long period non-progressive.

Of the forty-four cases ending in recovery, in twenty-three there was no medicinal treatment to which arrest of the disease could be attributed. In several of the twenty-three cases there was no medicinal treatment; in the remainder of the cases, the treatment consisted of simple tonics, palliatives of cough, or remedies to meet some other symptomatic indications. Of the thirty-one cases of non-progression of the phthisical disease without complete recovery, in fifteen there was no medication by which it might be supposed the disease had been controlled, and in several no medicinal treatment whatever. The two groups of cases—namely, those ending in recovery, and those becoming non-progressive without recovery—thus furnished about an equal proportion of those in which medicinal treatment was either wanting, or in no degree curative, the proportion in the first group being twenty-three of forty-four, and in the second group fifteen of thirty-one. In respect of hygienic or non-medicinal treatment, in some cases of both groups there was no change whatever in habits of life or other circumstance. In other cases there were changes involving improved hygienic conditions, but in a considerable number the changes were such that a potential influence could not be attributed to them. It is probably correct to say that the changes may have favored the recovery or the non-progression, but that they were inadequate to arrest the disease. In my work is introduced a condensed history of each of the seventy-five cases, which form the two groups now referred to.

A self-limited duration cannot be inferred from a single case, or from a very few cases, for this reason: the course and termination may have been affected by influences which are extrinsic, but not apparent. In order to obviate liability to error on this score, the number of cases must be sufficient to render it impossible, or vastly improbable, that in all such influences could have been overlooked. It is needless to say that the cases from which the inference of self-limitation is drawn must have been carefully observed and honestly recorded. Another requirement is essential—namely, there must be no room for distrusting the accuracy of the diagnosis. Assuming competency for observation and veracity, the diagnosis in each of the seventy-five cases is attested by the recorded histories, and it will be admitted that the number of cases is sufficiently large for the exclusion of error on the score of unrecognized extrinsic influences. The number of cases might be increased by the addition of those which have come under observation since 1875. This seems to be needless with a view to strengthen the conclusion respecting self-limitation. I therefore, submit, as substantiated by the clinical facts which I have cited, the following proposition—Pulmonary phthisis, in a certain proportion of cases, has a self-limited duration, the disease ceasing to exist after more or less progress of the local affection, all symptoms referable to the lungs disappearing, and recovery, as regards the general health, being complete. The disease is also self-limited in a certain proportion of cases in which lesions remain, giving rise to more or less of cough and expectoration, the persistence of these lesions not being incompatible with good general health and long duration of life.

It is an interesting fact that self-limitation is exemplified in the majority of fatal cases of phthisis. As is well-known, the disease, as a rule, advances not by a continuous progress, but by a series of successive invasions, separated by variable intervals. After each invasion, or, as it has been termed, tuberculous eruption, there is a temporary self-limitation of the disease. I will not venture on a discussion of the question whether this fact be sufficiently explained by the statement that each eruption of tubercles for a time exhausts the tuberculous cachexia, or whether the fact be owing to the production of successive broods of the bacilli tuberculosis. It suffices to state the clinical fact. The fact suggests a capital object in the treatment, namely, prevention of renewed invasion. The continuous advancement of the disease, as an exception to the rule, is the pathological feature of the so-called "galloping consumption," or phthisis florida.

In the cases ending favorably, which have been referred to as furnishing proof of a self-limited duration, the diagnostic symptoms and physical signs were so well marked, as to leave no room for doubt as to the existence of phthisis. From cases which have come under my observation, I have been led to believe that not very unfrequently phthisis ends by self-limitation without having advanced far enough for the diagnosis to be considered as positive. A patient has had for some time a slight cough, either dry or with a scanty expectoration; there has been some loss in weight, and the body heat is somewhat raised, with, perhaps, spitting of blood. These symptoms, taken in connection with the age of the patient, and, it may be, grounds for suspecting a congenital predisposition, point to a tuberculous affection. But examinations of the chest in such a case may fail to reveal distinct physical signs. Very likely the problem, as regards the physical diagnosis, is to determine whether at the summit of the chest on the right side there are abnormal signs, or only the normal points of disparity between the two sides. There may be found only a subcrepitant *râle*, or slight pleuritic rubbing, or an interrupted respiratory murmur at the summit on one side, without conclusive evidence of tuberculous solidification. Under these circumstances, the physician either commits his judgment to a diagnosis of incipient phthisis, or as is more probable, he reserves an opinion for further developments. After a short time all the pulmonary and general symptoms disappear. Now, if incipient phthisis have been diagnosticated, the physician concludes that the diagnosis was erroneous. He feels obliged so to conclude, in consequence of the common belief that phthisis does not thus commence and end from self-limitation. But it is highly probable that the diagnosis was correct. Phthisis existed and ended in its incipiency. It would be proper enough to distinguish these as cases of abortive phthisis. If I mistake not, all medical observers of much experience will admit that the foregoing sketch represents a class of cases not extremely rare. That they are not very rare is a fair inference from the frequency with which the traces of an old abortive phthisical affection are found in bodies dead with other diseases than phthisis.

A topic of practical importance is the bearing of self limitation on the prognosis in individual cases of phthisis. The analytical study of my collection of cases showed that, as a rule, in those which ended favorably from an intrinsic tendency, the tuberculous affection was moderate or small in amount, but that there are exceptions to this rule. All observers of much experience will agree that the prognosis in cases of phthisis is to be based more on the general condition of the patient than on the local symptoms and signs. To consider the elements of prognosis would be here out of place, even if time permitted. In general terms, the symptoms which denote tolerance of the phthisical affection, are those which indicate a favorable intrinsic tendency, and, on the other hand, pyrexia, progressive loss of weight, frequency of the heart's action, and anorexia, point to an opposite tendency. Of special importance, in a practical view, is the bearing of the doctrine of self-limitation on the conclusion to be drawn from observations, respecting the agency of therapeutic and hygienic measures in the treatment of cases of phthisis. How many and various are the remedies which have been supposed to have been sometimes curative in cases of this disease! Instances of their apparent curative power have been attested by honest observers. Making the fullest allowance for errors in diagnosis, I cannot doubt the credibility of more or less of these cases. Recovery has taken place under the employment of divers remedies; yet these remedies have so generally failed, that for the most part, they are now obsolete. The explanation of their apparent efficacy is to be found in the doctrine of self-limitation. The disease ended favorably, not from a specific influence of the remedies, but from an intrinsic tendency. This is not saying that the remedies may not have been, to a greater or less extent, serviceable. It may be laid down as a principle applicable to all diseases that, whenever experience has seemed to show success from treatment by a variety of remedies the efficient cause lies in the disease itself. This principle becomes more evident the more we become acquainted with the natural history of diseases. To accept this principle is not to disparage medicinal treatment. In certain cases of phthisis, as of other diseases, self-limitation is a factor co-working with curative measures, and, as perhaps may be added, sometimes effective, in spite of measures which obstruct its operation. On the other hand, when this factor is feeble or wanting, curative treatment is not likely to prove of much avail. Evidently, in drawing conclusions respecting the curative effect of remedies allowance is to be made for this factor. The extent of its co-operation, doubtless, differs much in different cases, in some being sufficient in itself, and in others either considerable, or moderate, or slight.

The doctrine of self-limitation bears on the climatic and other measures entering into the hygienic treatment of cases of phthisis with not less force than on the employement of drugs. As regards climate, is there a practical theorem more perplexing to the practitioner of medicine than that of selecting the best resorts for phthisical patients, provided the selection be made on the basis of an impartial consideration of the reported results of climatic agencies in different situations? Underlying the exaggerations on the one hand, and on the other hand the depreciations of particular climatic resorts, founded on the different results in a few cases, is the factor of unknown power, self-limitation, the existence of which is generally ignored. Here is the explanation, at least in part, of the discrepancies of testimony concerning the results of climatic influences in different situations.

The extent of influence attributable to self-limitation in phthisis is by no means as yet ascertained. There is ample room for observation bearing on this point of inquiry. Impressed with the importance of clinical studies having this direction, I cannot forbear the remark that they promise more in the way of practical utility than has hitherto been derived from the discussion of the histologico-pathological questions which, of late years, have engrossed so much attention and occupied so large a space in medical literature.

